# Maternal Confidence and Parenting Stress of First-Time Mothers in Taiwan: The Impact of Sources and Types of Social Support

**DOI:** 10.3390/healthcare10050878

**Published:** 2022-05-10

**Authors:** Hsin-Hui Huang, Tzu-Ying Lee, Xin-Ting Lin, Hui-Ying Duan

**Affiliations:** 1 Department of Infant and Child Care, National Taipei University of Nursing and Health Sciences, Taipei 112303, Taiwan; hsinhui@ntunhs.edu.tw (H.-H.H.); a345810@gmail.com (X.-T.L.); 2 School of Nursing, National Taipei University of Nursing and Health Sciences, Taipei 112303, Taiwan; tzuying@ntunhs.edu.tw

**Keywords:** parenting stress, social support, maternal confidence, first-time mothers, sources of social support, types of social support

## Abstract

The adjustment process to becoming a mother is affected by culture. However, earlier studies have not clarified the relationship between parenting stress, social support, and maternal confidence in non-Western women. This study examined the associations between different types and sources of social support, maternal confidence, and parenting stress experienced by first-time mothers. The sample consisted of first-time mothers with a child under one year of age in northern Taiwan, and a total of 205 valid questionnaires were collected. The results supported the stress-buffering hypothesis, which suggests that social support reduces the adverse effect of stress on maternal confidence. Although previous studies have suggested that spouses and maternal relatives are critical in supporting first-time mothers’ transition into their new roles, each source did not show a mediator effect in our study. The beneficial effect of social support was found only when all social network members collectively participated. Regarding the types of social support, only appraisal support had a significant mediator effect; no effect was found for emotional, instrumental, or informational support. These findings add to our understanding of how different types and sources of social support play a role in helping first-time mothers adapt.

## 1. Introduction

Becoming a new mother is accompanied by many life disruptions. A meta-analysis of qualitative studies on new mothers in Australia and the U.S. resulted in five categories of disruptions: commitments, daily life, relationships, self, and work [1]. Almost every aspect of a woman’s life is influenced. Challenges and difficulties arise with new responsibilities that cause a high prevalence of distress for new mothers. In one study taking a broader classification of postnatal distress, around 30% of Australian new mothers reported having symptoms of anxiety, stress, and depression [2]. Another meta-analysis revealed that 19.2% of women from developed countries (primarily from Eastern cultures) had a depressive episode during the first three months postpartum [3]. Some stressors include a lack of sleep, adjusting and planning the focus of personal life, infant care, and financial problems. These stress factors can affect the physical and mental health of the mother [4], the mother–infant interactions, and the development of the infant [5]. Mothers who lack a sense of control under high-stress conditions can feel inadequate in their ability to care for their baby; the stressful feeling of parenting is closely related to low maternal confidence [6].

Maternal confidence refers to a mother’s perception that she can understand and meet her baby’s needs and has the skills and abilities to care for the baby [7,8]. Maternal confidence is an indicator of a successful transition to motherhood during parental role adjustment [7,9]. A mother’s lack of confidence not only affects her self-concept and physical and mental health, but may also make her less sensitive to her parental roles and damage parent–child relationships [9,10].

Social support is a critical predictor of and positively affects maternal confidence [11,12,13]. Social support refers to the emotional care, instrumental assistance, information, and affirmation provided by social network members when an individual needs support [14]. According to Leahy-Warren, et al. [15] and Cohen and Wills [16], the four types of support can be defined as follows: Emotional support refers to the care, listening, and comfort offered by others. Instrumental support refers to financial, material, or labor assistance. Informational support refers to the knowledge or advice provided by other people to solve problems. Appraisal support is the feeling of affirmation and respect given by others.

In the postpartum stage, social support provides first-time mothers with role models and resources to learn society’s accepted way of mothering and cope with difficulties and stressful events [11]. Those with lower social support are more likely to encounter difficulties in role adaptation [17] and are more vulnerable to depressive and anxiety symptoms [18]. Findings from countries in East and Southeast Asia have shown that if postpartum women are single, their psychological well-being and maternal confidence are lower than that of married women [9,19].

As social support plays an essential role in the mental health of individuals, several attempts have been made to examine the differential impacts of various types and sources of social support. Postpartum women in different cultures, such as those from Iran, Ireland, and Canada, all consider the support of their spouses and their mothers to be the most crucial factor in building confidence [11,15,20]. Research in Japan has shown that the fewer resources available to mothers, the more unfavorable the connection between mother and baby [21]. The types of support may also be different due to the healthcare system and culture. For example, mothers from China and Singapore have indicated receiving more emotional and appraisal support than informational and instrumental support [13,22]. At the same time, research in Ireland has shown that first-time mothers receive less emotional and appraisal support [15].

There has been extensive research on social support, parenting stress and/or depression, and confidence/self-efficacy among postpartum women [23,24]; however, the literature is predominantly based on women in Western contexts. Furthermore, the results are primarily based on data from women of mixed parity or sub-groups of women, such as those with older children or children with special needs; this makes it difficult to draw conclusions about first-time mothers in early postpartum, who are likely to have distinct experiences. Finally, many studies have only examined the pathways between these three factors in isolation, meaning a causal model of their relationships is currently lacking.

According to the stress-buffering hypothesis, social support directly enhances positive emotions and self-worth and can improve an individual’s mental health; social support can also reduce the impacts of potentially stressful situations and indirectly promote individual mental health [16,25]. The first purpose of this study was to test whether social support plays a mediator role between parenting stress and maternal confidence. Furthermore, the adjustment process to becoming a mother is affected by culture. Since interpersonal relationships are highly valued in Eastern culture [26,27], the sources of support felt by postpartum women may be more diverse than women in Western culture, and the types of support may also vary from source to source. Therefore, the second purpose of this study was to explore the sources and types of social support experienced by first-time mothers in a non-Western context and to explore whether each type and source of support weighs differently in mediating the relationships between parenting stress and maternal confidence.

## 2. Materials and Methods

### 2.1. Participants

The participants met the inclusion criteria for being first-time mothers with a full-term infant under one year of age without a known medical condition. They were recruited at parent–child play centers (*n* = 73), well-child clinics (*n* = 64), and infant care centers (*n* = 68) in Taipei, Taiwan; there were 205 valid questionnaires. The mean age of the participants was 32.80 years (range 21–42, *SD* = 4.07); they predominantly lived in urban regions of northern Taiwan and the mean age of their infants was 7.25 months (range 1–12, *SD* = 3.11). There were 174 mothers (84.90%) with a university undergraduate level of education or above, and 51.2% were full-time caregivers (including those on a parental level). According to statistics from the Ministry of the Interior of Taiwan in 2021, 81.96% of women between 20 to 39 years of age who live in Taipei have a university undergraduate education or above, and the average age of first childbirth is 31.09 [28]. Thus, the sample in this study is fairly representative of the population.

### 2.2. Measures

#### 2.2.1. Maternal Confidence Questionnaire

This study used the Maternal Confidence Questionnaire (MCQ) to measure the confidence of first-time mothers in their parenting ability and ability to understand the needs of their babies [29]. The MCQ has 14 questions and applies a five-point Likert scale ranging from 1 (never) to 5 (always). After obtaining the original author’s consent [8], this study translated the MCQ into Mandarin for use. A preliminary analysis found that the relevance of Questions 10 and 12 to the total score after reverse scoring was too low (*r* = 0.18 and −0.01); therefore, both questions were removed, leaving 12 questions in the questionnaire with a Cronbach’s α value of 0.85.

#### 2.2.2. Social Support Rating Scale

This study obtained permission from the author to use the Social Support Rating Scale (SSRS) [30]. The SSRS was modified from the Interpersonal Support Evaluation List developed by Cohen and Hoberman [31] to make it suitable for the mothers’ responses. The SSRS adopts a four-point Likert scale (from 1 = never to 4 = always) and includes four types of social support, including emotional, informational, appraisal, and instrumental support, with four questions for each type of support, for a total of 16 questions. Sample questions included “They will comfort me when I need them” (emotional), “They will provide suggestions to solve my problems” (informational), “They make me feel important” (appraisal), and “They will share my chores when I feel physically or psychologically uncomfortable” (instrumental).

Participants indicated the degree of assistance they subjectively felt from four sources: spouses, maternal relatives, in-laws, and friends. The Cronbach’s α values were 0.89–0.97 for each type and source of social support and 0.97 for the entire scale.

#### 2.2.3. Parenting Stress Index: Short Form

The Parenting Stress Index: Short Form-Chinese version (PSI/SF-C) [32] was translated from the original version developed by Abidin [33] and used to assess the pressure experienced by first-time mothers in their motherhood role. A five-point Likert scale (from 1 = strongly disagree to 5 = strongly agree) was adopted for the PSI/SF-C, which included the three subscales of parental distress (PD), parent–child dysfunction interaction (PCDI), and child with difficulties (DC), with 12 questions each, for a total of 36 questions. The correlation of each item in the subscale was above 0.30, and the factoring load was greater than 0.30 [32]. According to the original version of the PSI, scores between the 15th and 80th percentile are within the normal range, and scores above the 90th percentile are considered borderline clinically significant [33].

### 2.3. Data Collection

Prior to data collection, the institutional review board of the National Taipei University of Nursing and Health Sciences approved the study protocol (date of approval: 2017/1/23). The researchers first contacted the parent–child play centers and well-child clinics. With the verbal consent of the agency directors, the researchers visited the agencies and approached potential participants to introduce the study, including the purpose, process, and research ethics. If the subject met the sampling criteria and verbally consented to participate, an anonymous paper questionnaire was filled out on the spot. In addition, the researchers approached infant care centers to ask for help issuing questionnaires to parents of infants under one year of age and returning them in sealed envelopes.

### 2.4. Data Analysis

Descriptive and correlation analyses were employed using the SPSS version 20 software. Model 4 of the Hayes PROCESS macro v4.0 for SPSS [34] was adopted to test the single mediation model of the overall social support and the parallel mediation models of the support source and the support type. A bootstrap sampling method using 5000 iterations was employed to test the abovementioned mediation models. Preliminary analyses were conducted to test the relationships between sample characteristics (i.e., child age, maternal age, education level, and working status) and the main variables. Since the numbers of mothers with high school or below and college education were relatively small compared to the other two categories, the education levels were collapsed into two groups (college and below, university and above). The results showed that only child age was positively correlated with maternal confidence; therefore, the mediation analyses were adjusted for infant age in months.

## 3. Results

### 3.1. Descriptive Analyses

Table 1 and Table 2 present the sample characteristics and descriptive statistics of the research variables. The average parenting stress score was 78.19 (range 35–145). The subscale with the highest average score was parental distress, followed by difficult child and parent–child dysfunctional interaction. Based on the percentile rank, 50 mothers had a low stress level (below the 15th percentile), 22 had a high stress level (above the 80th percentile), and the remaining 133 were in the normal range. The average maternal confidence score was 49.74 (range 32–60), showing reasonable confidence in motherhood. The average overall social support score was 194.51 (range 108–256). In terms of sources, maternal relatives scored the highest, followed by spouses and friends, then in-laws. Regarding the types of support, the appraisal and emotional support scores were higher, while the informational and instrumental support scores were slightly lower.

### 3.2. Differences between Sources and Types of Social Support

A repeated measures ANOVA showed a significant difference in the scores of the four support sources (*F* (3, 609) = 62.02, *p* < 0.001, ƞ^2^ = 0.23). As indicated by post hoc comparisons, with the exceptions of two groups (spouses and maternal relatives, spouses and friends), the remaining pairs of support sources had significant differences (*p* < 0.001). There were also significant differences in the scores of the four types of social support (*F* (3, 609) = 40.79, *p* < 0.001, ƞ^2^ = 0.18). Post hoc comparisons did not show a significant difference between emotional and appraisal support, but significant differences between the other types of support (*p* < 0.05).

### 3.3. Correlational Analyses

The relationship between overall social support, parenting stress, and maternal confidence was viewed using a Spearman rank correlation, which showed that parenting stress and overall social support (*r* = −0.350, *p* < 0.001) and parenting stress and maternal confidence (*r* = −0.368, *p* < 0.001) were negatively correlated. There was a positive correlation between maternal confidence and overall social support (*r* = 0.301, *p* < 0.001).

The relationship between different sources and types of social support and parenting stress is shown in the upper part of Table 3. The supports provided by the four sources were all related to lower parenting stress (*r* = −0.210 to −0.262), as were the four types of social support (*r* = −0.202 to −0.357). When examining each type of social support provided by different sources, the negative correlations between social support and parenting stress held, except for the informational support from in-laws and friends.

The bottom half of Table 3 shows the relationship between the sources and types of social support and maternal confidence. All sources (*r* = 0.180 to 0.258) and types (*r* = 0.216 to 0.312) of social support had a significant positive correlation with maternal confidence. When examined, the types of support from each source were positively correlated with maternal confidence, except for the informational support of in-laws and friends.

### 3.4. Tests of Mediation

**Figure 1:**
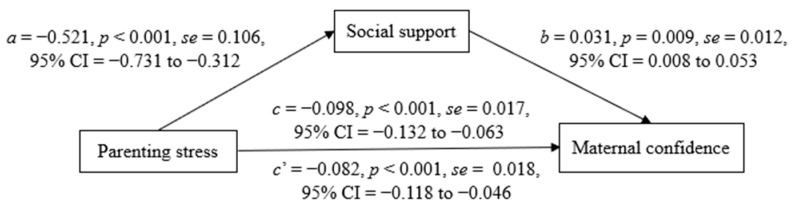
Effects of parenting stress on maternal confidence with the mediator of overall social support, adjusted for infant age by month (*N* = 205).

First, we tested the effect of overall social support as a single mediator (Figure 1). Parenting stress was negatively associated with overall social support (*a* = −0.521, *p* < 0.001), overall social support was positively associated with maternal confidence (*b* = 0.031, *p* = 0.009), and parenting stress was negatively associated with maternal confidence (*c* = −0.098, *p* < 0.001). After including overall social support, while parenting stress was still negatively associated with maternal confidence (*c*’ = −0.082, *p* < 0.001), its effect was reduced, and the variance explained by the model was 21.98%. The indirect mediating effect calculated by the Sobel’s product of coefficients (a*b) was −0.016 (*se* = 0.008, 95%CI = −0.035 to −0.002), which shows a significant partial mediating effect; the mediating effect size (a*b/c) was 16.26%.

**Figure 2:**
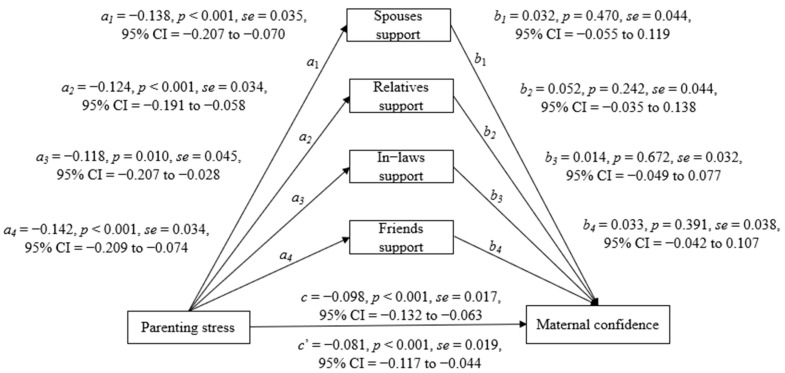
Effects of parenting stress on maternal confidence with the mediator of sources of social support, adjusted for infant age by month (*N* = 205).

The second mediation analysis tested four sources of social support as mediating variables for the parallel mediating analysis (Figure 2). Parenting stress was negatively associated with spouse support (*a*_1_ = −0.138, *p* < 0.001), maternal relatives support (*a*_2_ = −0.124, *p* < 0.001), in-laws support (*a*_3_ = −0.118, *p* = 0.010), and friends support (*a*_4_ = −0.142, *p* < 0.001). Parenting stress was also negatively associated with maternal confidence (*c* = −0.098, *p* < 0.001). However, the four sources of social support were not related to maternal confidence (*b*_1_~*b*_4_ = 0.014 to 0.052, *p* = 0.242 to 0.672). This result showed that the individual sources of social support did not have a mediating effect.

**Figure 3:**
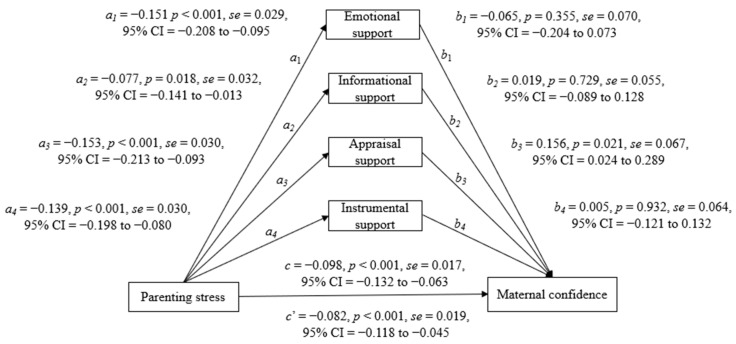
Effects of parenting stress on maternal confidence with the mediator of types of social support, adjusted for infant age by month (*N* = 205).

The third mediation analysis tested the four types of social support as mediating variables for a second parallel mediating analysis. As indicated in Figure 3, parenting stress was negatively associated with emotional support (*a*_1_ = −0.151, *p* < 0.001), informational support (*a*_2_ = −0.077, *p* = 0.018), appraisal support (*a*_3_ = −0.153, *p* < 0.001), and instrumental support (*a*_4_ = −0.139, *p* < 0.001), as well as maternal confidence (*c* = −0.098, *p* < 0.001). Although the four types of social support and parenting stress jointly predicted maternal confidence, only parenting stress (*c*’ = −0.082, *p* < 0.001) and appraisal support (*b*_3_ = 0.156, *p* = 0.021) were related to maternal confidence, while the other three types of support were not related (*b* = −0.065 to 0.019, *p* = 0.355 to 0.932). As the effect of *c*’ was reduced, it meant that the appraisal support could partially mediate the effect of parenting stress on maternity confidence; the explained variance was 21.98%. The indirect mediating effect (a*b) of appraisal support was −0.024 (*se* = 0.013, 95%CI = −0.051 to −0.001), showing a significant partial mediating effect; the mediating effect size (a*b/c) was 24.44%.

## 4. Discussion

The results showed that, on average, mothers perceived a low to normal level of stress [32] and a moderate to high degree of support and maternal confidence. In terms of support, mothers perceived themselves as receiving more help in emotional and appraisal aspects of support, and the significant sources of support came from spouses and maternal relatives. Friends also provided emotional and appraisal support levels that were almost comparable to those of spouses and maternal relatives. These results corroborate the findings of the limited previous work on the Taiwanese population. In a study by Liu [35], 13.3% of mothers with an infant under one year had postpartum depression, compared to 10.73% of our sample who had a high stress level. Another study of working mothers with a child younger than two years found that mothers received more emotional and less instrumental and informational support [36].

The low to normal stress level and moderate to high degree of maternal confidence suggest that most of our sample were well adapted to motherhood. These results may be attributable to the fact that most mothers w in the second half of their infant’s first year. After accumulating care experiences, mothers typically become more confident in identifying the needs of their children and providing appropriate responses while adjusting to the motherhood role and establishing the parent–child relationship [22]. The sample in this study may thus have passed the stage of role change and high stress.

The results of the first mediation analysis support the buffering hypothesis of social support [16]; that is, social support networks can directly enhance the confidence of first-time mothers and indirectly reduce the negative effect of stress on confidence. This result is generally in line with previous findings that social support has a beneficial effect on well-being [16]. Being a mother means changes in all aspects of life, thus creating stress and anxiety when faced with unknown challenges [37,38]. Although part of our sample could have passed the stage of role change, all of them still needed to adapt to meet the constantly changing needs of their children within the first year of rapid development. Social networks help first-time mothers at the time of change, support them with problems, and prepare them mentally for what may come [39], thus mitigating the adverse effect of stress on mental health.

However, the second mediation analysis suggested that individual sources of social support did not have a mediating effect. Past studies have found that being single and having fewer sources of support available during pregnancy or after delivery is detrimental to new mothers’ physical and psychological well-being and their bond with their infants [7,9,21]. The above argument may be more appropriate in Eastern cultures that value interpersonal relationships. Since the support needs and sources of new mothers evolve as they go through the stages of motherhood [40], all supporters are essential, and each plays complementary roles in the social network. Recent evolutionary anthropological research provides another perspective which argues that for mothers raising newborns, what matters most is the quality and quantity of social support rather than who provides the support, as the latter is subject to vary by culture [41]. This viewpoint is worth considering, especially in a world with more diverse and non-traditional forms of families.

The third mediation analysis revealed that only appraisal support had a significant partial mediating effect. This result suggests that being affirmed and recognized as appropriately playing the role of a new mother may be a crucial factor in buffering the negative impact of parenting stress on the maternal confidence of first-time mothers. One explanation may be that half of the sample cared for their infants full-time; thus, receiving recognition for competent performance as new mothers fosters a sense of satisfaction. The appraisal support score was also the highest among the four types of support, which is different from the findings of previous studies in other cultures and women in different postnatal stages [15,42]. The inconsistency may be because our sample had mastered infant care chores and thus received more affirmation. Therefore, information and instrumental support become less critical after a few months postnatally.

The generalizability of these results is subject to certain limitations. First, the study used a convenience sample that may be prone to select participants with specific characteristics. For example, most of our participants were recruited from public places when they took their infants to play with others, get a physical check, or get vaccinations. Hence, our participants may tend to seek out external support voluntarily and be well adjusted to motherhood. In addition, because the measures were self-reported, it was impossible to infer causality in the relationship among variables. Finally, this study only controlled one confound in the models. Other potential confounding factors need to be pursued in future studies.

The precise mechanism of social support in non-Western samples of first-time mothers remains to be elucidated. Further research could explore how each type of social support plays a specific role in the different stages of motherhood. This knowledge would help tailor social policy to support first-time mothers more effectively, especially in low-fertility societies with fewer mother models.

One of the findings that caught our attention was the informational supporting role of spouses. Although it was perceived as the lowest among the sources, spouses’ informational support was related to lower parenting stress and higher maternal confidence. This finding suggests that although first-time fathers did not have much information to solve childcare problems, their partners still valued their input. Parenting programs that encourage mothers and their spouses to attend together and adjust the content to focus on co-parenting may be more effective in assisting both parents smoothly transition to parenthood and form partnerships in raising their infant together.

Being a new mother is one of the most challenging experiences that can occur in adulthood. This study demonstrated that although each source provides a different level of support, the buffering effect of social support for first-time mothers is only valid when all social network members participate. This study also found that not all types of support exert the same effect. Affirming primipara’s capabilities in their new parenting role is crucial to helping women successfully transition to motherhood.

## Figures and Tables

**Table 1:** Sample characteristics (*N* = 205).

Demographic	Frequency (*n*)	Percentage (%)	Mean (*SD*)	Range
Child age (months)			7.25 (3.11)	1–12
Mother age (years)			32.80 (4.07)	21–42
Education level				
High school or below	14	6.8		
College	17	8.3		
University	127	62.0		
Graduate	47	22.9		
Working status				
Yes	100	48.8		
No (including maternal leave)	105	51.2		

**Table 2:** Descriptive statistics of social support, parenting stress, and maternal confidence (*N* = 205).

Variables	Mean (*SD*)	Spouses Mean (*SD*)	Maternal Relatives Mean (*SD*)	In-Laws Mean (*SD*)	Friends Mean (*SD*)
Parenting stress					
Parental distress	29.53 (7.97)				
Parent–child dysfunctional interaction	21.49 (6.71)				
Difficult child	27.22 (8.65)				
Total	78.19 (19.86)				
Maternal confidence	49.74 (5.55)				
Social support					
Emotional	49.90 (8.59)	13.37 (2.91)	13.42 (2.71)	10.05 (3.71)	13.06 (2.67)
Informational	45.58 (9.24)	10.47 (3.44)	12.55 (2.97)	10.59 (3.37)	11.99 (3.26)
Appraisal	50.37 (9.07)	13.34 (2.93)	13.18 (2.78)	10.82 (3.53)	13.02 (2.59)
Instrumental	48.65 (8.83)	13.94 (2.53)	13.27 (2.91)	10.56 (3.72)	10.89 (3.16)
Total	194.51 (31.64)	51.11 (10.15)	52.42 (9.76)	42.03 (12.86)	48.96 (10.02)

**Table 3:** Spearman’s rho between parenting stress, maternal confidence, and social support (SS) by types and sources (*N* = 205).

Variables	Total SS	Spouses	Maternal Relatives	In-Laws	Friends
Parenting stress vs.					
Emotional support	−0.357	−0.276	−0.275	−0.216	−0.265
Informational support	−0.202	−0.169	−0.144	−0.106 ^a^	−0.119 ^a^
Appraisal support	−0.330	−0.250	−0.263	−0.208	−0.291
Instrumental support	−0.330	−0.245	−0.254	−0.190	−0.220
Total	−0.350	−0.262	−0.257	−0.210	−0.253
Maternal confidence vs.					
Emotional support	0.249	0.245	0.197	0.155	0.186
Informational support	0.216	0.186	0.165	0.115 ^a^	0.136 ^a^
Appraisal support	0.312	0.278	0.332	0.171	0.238
Instrumental support	0.279	0.170	0.219	0.186	0.242
Total	0.301	0.248	0.258	0.180	0.232

^a^*p* > 0.05.

## Data Availability

The data presented in this study are available on request from the first author. The data are not publicly available due to ethical restrictions.
